# Computer aided volumetric assessment of orbital structures in patients with Graves' orbitopathy: correlation with serum thyroid antiperoxidase antibodies and disease activity

**DOI:** 10.1007/s10792-023-02745-8

**Published:** 2023-06-20

**Authors:** Laura Paniagua, Manuel F. Bande, Jose M. Abalo-Lojo, Francisco Gonzalez

**Affiliations:** 1 Service of Ophthalmology, Complexo Hospitalario Universitario de Ferrol, Av. da Residencia, S/N, 15405 Ferrol, Spain; 2 Service of Ophthalmology, University Hospital of Santiago de Compostela, Ramon Baltar S/N, 15706 Santiago de Compostela, Spain; 3 CIMUS, University of Santiago de Compostela, Avd. Barcelona S/N, 15782 Santiago de Compostela, Spain; 4 Instituto de Investigacion Sanitaria de Santiago de Compostela (IDIS), Santiago de Compostela, Rua da Choupana S/N, 15706 Santiago de Compostela, Spain; 5 PhD student, Universidade de Santiago de Compostela, Santiago de Compostela, Spain

**Keywords:** Graves' orbitopathy, Graves-Basedow, Thyroid, Thyroid hormones, Orbit

## Abstract

**Introduction:**

Graves' disease is an autoimmune disorder. Goiter and Graves' orbitopathy are frequently seen clinically. It would be helpful for the diagnosis, grading, prognosis, and treatment of this condition if it was possible to find serum biomarkers to establish a connection between the plasma levels of these compounds and orbital changes.

**Methods:**

A retrospective study was performed by revising the medical records of 44 patients with Graves' orbitopathy and 15 controls. The Osirix software (Pixmeo, Geneva, Switzerland) was used for manual orbital measurements. Plasma levels of Graves' orbitopathy substances were obtained in the analytical review of the patients.

**Results:**

A greater muscle volume was observed in patients with Graves' orbitopathy in relation to the control group (*p* < 0.001). The clinical activity score (CAS) was associated to total muscle mass (*p* = 0.013) and retrorbital fat (*p* = 0.048). Our results indicated a direct relationship between serum concentrations of anti-thyroid peroxidase antibodies and inferior rectus thickening (*p* = 0.036); however, we did not observe a positive correlation between other muscle volumes and serum concentrations of various thyroid-related substances.

**Conclusions:**

This study is the first that uses Osirix measurement software to manually assess orbital features in patients with Graves' orbitopathy. These measurements were compared to the outcomes of tests performed in a laboratory. Among several serum biomarkers, anti-thyroid peroxidase appears to be a reliable biomarker that correlates positively with inferior rectus muscle thickness in patients with thyroid eye disease. This may help to improve the management of this disease.

**Supplementary Information:**

The online version contains supplementary material available at 10.1007/s10792-023-02745-8.

## Introduction

Graves’ disease (also called Graves-Basedow Disease or *GD*) is an autoimmune disorder that causes thyroid abnormalities that manifest as hypo- or hyperthyroidism; a small percentage of patients may be euthyroid. Clinically, goiter, Graves' orbitopathy (GO), and sometimes localized dermopathy known as *pretibial myxedema* may occur. Other signs and symptoms include weight loss, irritability, muscle weakness, insomnia, tachycardia, diarrhea, and poor heat tolerance [1].

While there have been many advances in GO in terms of molecular immunology, less attention has been paid to quantifying retroorbital fat and extraocular muscle volume. Historically, muscle volume measurements were made using imaging tests in healthy participants and patients with oculomotor palsy [2] and/or myositis [3, 4]. Muscle dynamics were also studied [5, 6]. However, studies quantifying the size or volume of orbital structures in GO are scarce. Likewise, the few studies that used MRI, and CT [7, 8] did not attempt to correlate their results with serum biomarkers.

Patients with GO demonstrate specific serum constituents—including thyroid hormones, antibodies, and proteins—that activate thyroid cell receptors or cytokines [9]. The inflammatory activity present in GO alters the morphology of orbital structures; consequently, there may be a correlation between the serum levels of these substances and orbital alterations. Establishing such relationships may help diagnose, grade, prognosticate, and treat this disease.

The objective of the present study was to quantify the dimensions of orbital fat, extraocular muscles and optic nerve and look for potential correlations between these values and serum levels of different substances related to GD and GO.

## Methods

We included 38 patients being followed within the Oculoplastic and Orbit Unit of the Ophthalmology Service of Complejo Hospitalario Universitario de Santiago de Compostela. All participants were diagnosed with GD and orbitopathy with orbital imaging test results.

The 15 controls were patients who underwent MRI scans for other reasons unrelated to orbital problems, had no history of orbital disease, and had no thyroid-related abnormalities. All patients and controls were white Caucasian to avoid interracial differences in orbital anatomy [10]. Those subjects with history of orbital surgery, abnormal orbital structures secondary to trauma or tumors, high myopia or other orbital abnormalities were also excluded.

Before study entry, all patients underwent a complete ophthalmologic examination performed by the same ophthalmologist that included visual acuity, intraocular pressure (Perkins Tonometer, Haag-Streit Diagnostics, Harlow, England), exophthalmometry (Hertel exophthalmometer, Richmond Products, Inc, Albuquerque, USA), ocular motility examination, assessment of eyelid position, slit lamp examination, and fundus ophthalmoscopy. We additionally evaluated the activity and severity of orbitopathy according to CAS, and NO-SPECS scales using the information recorded closest to the imaging study used to perform the measurements. Serum values of thyroid stimulating hormone (TSH), free T3, free T4, thyroid stimulating immunoglobulin (TSI), anti-thyroid peroxidase (TPO), thyroglobulin, anti-thyroglobulin antibodies and erythrocyte sedimentation rate (ESR) were obtained from laboratory test made as close as possible to the imaging studies.

The MRI equipment used for the analysis were the Ingenia 1.5 T CX (Philips Medical Systems, Nederland B.V.) and the Philips Ingenuity CT (Philips Medical Systems, Nederland B. V.). We used consecutive slices that included the entire orbit, with an inter-slice separation of 3 mm. The size of the images was 512 × 512 pixels. Image analysis was performed manually using commercially available software (OsiriX Lite 8.0, Pixmeo, Geneva, Switzerland). Using different software tools and manual segmentation techniques, the regions of interest were delimited using anatomical landmarks. For orbital measurements, the orbital septum and orbital rim (frontal bone, frontozygomatic suture, and anterior lacrimal crest) were selected as the anterior limit, while the posterior limit was determined by the bony contour formed by the frontal bone, lesser wing of the sphenoid, greater wing of the sphenoid, ethmoid bone, lacrimal bone, palatine bone, zygomatic bone, and maxillary bone, up to the optic canal entrance in the lesser wing of the sphenoid.

For estimating the volume of muscles and optic nerve, coronal sections of the images were used, with the perimeter of the muscle section being defined in all slices. Integration of these sections resulted in the total muscle volume. Due to the intimate anatomical relationship between the superior rectus muscle and levator palpebrae superioris muscle, both muscles were considered as a single muscle.

To measure the length of the optic nerve, transverse sections were selected using the "Size" function in Osirix, which measures the distance between manually selected points (in cm) even if they are in different slices. The complete optic nerve was visualized in the selected image section, and its length was measured from the posterior aspect of the eyeball to the optic canal at the apex of the orbit near the optic chiasm. This measurement actually indicates the distance from the posterior pole of the eyeball to the orbital apex, rather than the real length of the optic nerve, as it is normally slightly curved. Thus, this measurement represents the degree of optic nerve stretching rather than its actual length, but for the sake of simplicity, we refer to it as "optic nerve length" in this study.

**Fig. 1:**
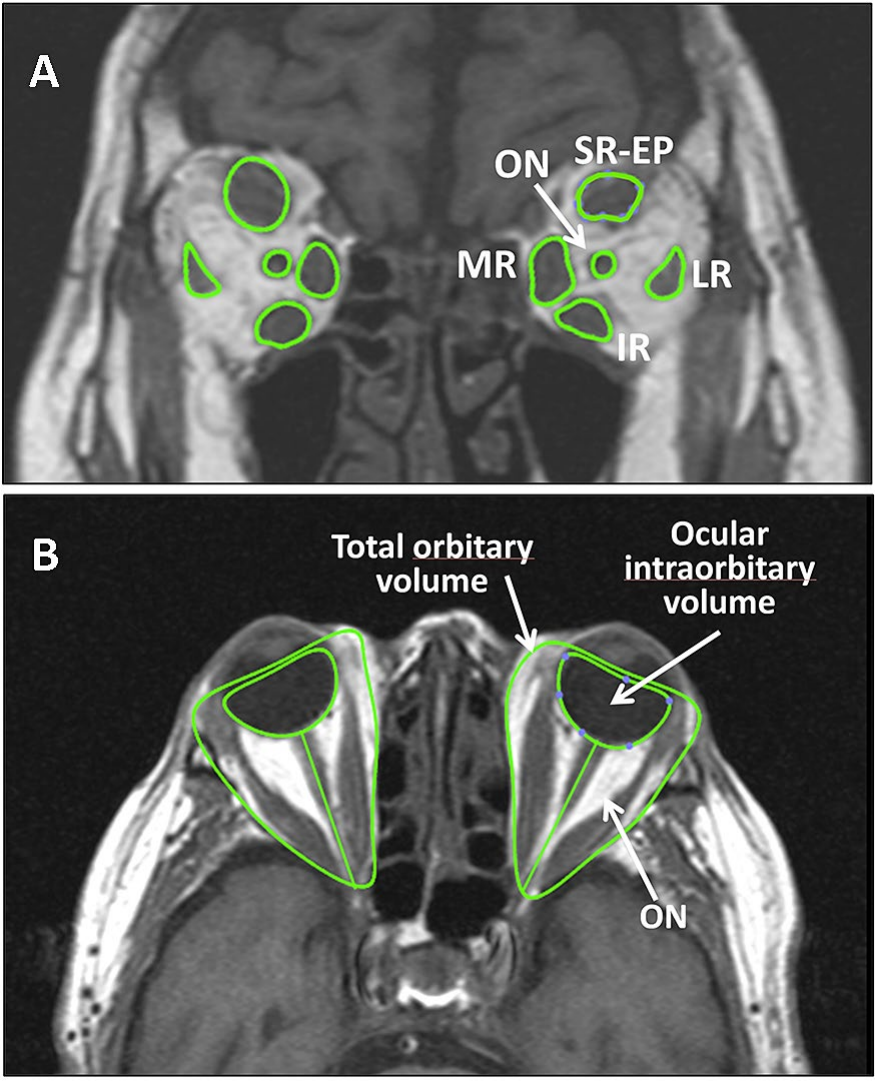
MRI image of a patient, in which the regions of interest are outlined. The images were transferred in DICOM format to Osirix Lite software in order to calculate the volume of the studied orbital structures. **A**: SR-EP: Superior rectus -Elevator of the eyelid complex; LR: Lateral rectus; IR: Inferior Rectus; MR: Medial Rectus; ON: Optic nerve. **B**: Delimitation of total orbitary volume and intraorbitary portion of the eyeball are indicated. The total orbital fat was assumed to be the total orbital volume after subtracting the intraorbital portion of the eyeball as well as the volume of extraocular muscles (SR-EP, LR, IR, MR) and optic nerve

The volume of orbital fat was estimated by subtracting the volumes of structures that predominantly occupy the orbit (muscles, optic nerve and eyeball) from the total orbital volume. All volumes were expressed in cm^3^ (Fig. 1). All participants provided written informed consent, according to the Declaration of Helsinki, prior to participation in the study, which was approved by the Clinical Research Ethics Committee of Galicia (Registration 2016/232).

All analyses were performed using the statistical program R v2.15.1 (The R Project for Statistical Computing, www.r-project.org). Scatter diagram matrix was created using the GGally package. Descriptive statistics were expressed as absolute frequencies and percentages. Mean, median, minimum, and maximum values were used for quantitative analysis.

Between-group differences were analyzed using the nonparametric Kruskal–Wallis and Mann–Whitney U tests according to the number of factors involved. We used nonparametric tests because of the relatively small study cohort and the non-normal data distribution. Spearman’s correlation coefficient (R) was used to evaluate the relationship between orbital and serum tests measurements made in the same patient. Correlations were made between total muscle mass (MM, added volumes of all muscles in both eyes of each patient) and serum test parameters (TSH, TSI, TPO, free T4, free T3, thyroglobulin (Tg), anti-Tg antibodies, and ESR), and exophthalmos. Correlations between total orbital fat (the sum of the fat volumes from each participant’s two orbits) and serum test parameters were also analyzed. *p* values ≤ 0.05 were considered statistically significant.

## Results

The 38 patients had a mean age of 48 years and a standard deviation of 15.4 years, with a range of 19–79 years, comprising 37% (14/38) males and 63% (24/38) females. The 15 controls were matched to patients by sex and age to ensure that the groups were statistically similar at baseline (Chi-squared Test, Mann–Whitney test, *p* > 0.05). This group consisted of 5 males (33%) and 10 females (67%), with a mean age of 47.2 years (range 22–72).

Diplopia was present in 25.6% of the patients and 75.6% had exophthalmos (≥ 20 mm) on examination. The patients’ mean exophthalmos was 21 mm for the right eye (OD; range 26–15 mm) and 21 mm for the left eye (OS; range 26–15 mm). Diplopia was related to increased muscle volume; this relationship was statistically significant for the superior (Mann–Whitney test, *p* = 0.031) and medial rectus muscles (Mann–Whitney test, *p* = 0.037) in the patients group. Also, the existence of exophthalmos was related to fat volume (Mann–Whitney test, *p* = 0.029), while no statistically significant relationship was observed between exophthalmos and muscle volume or optic nerve length. No controls had diplopia or exophthalmos at the time of examination (Table 1s). When we compared the mean muscle volume between patients and controls, all extraocular muscles [medial rectus (MR), inferior rectus (IR), lateral rectus (LR), and superior rectus (SR)] were significantly thicker in patients than in controls (Table 1).

**Table 1:** Volume of the extraocular muscles (cm^3^). SR: superior rectus; MR: medial rectus; LR: lateral rectus; IR: inferior rectus; MM: total muscle mass. The rightmost column shows the comparison between controls and patients

Muscle volume							
Group							
	Controls			Patients			Mann–Whitney, *p*
	Median	Maximum	Minimum	Median	Maximum	Minimum	
SR	0.23	0.37	0.09	0.41	1.50	0.15	< 0.001
MR	0.26	0.38	0.10	0.47	1.14	0.23	< 0.001
LR	0.20	0.28	0.10	0.47	0.89	0.25	< 0.001
IR	0.31	0.52	0.11	0.56	1.94	0.18	< 0.001
MM	2.14	3.23	0.81	3.88	10.46	2.08	< 0.001

**Fig. 2:**
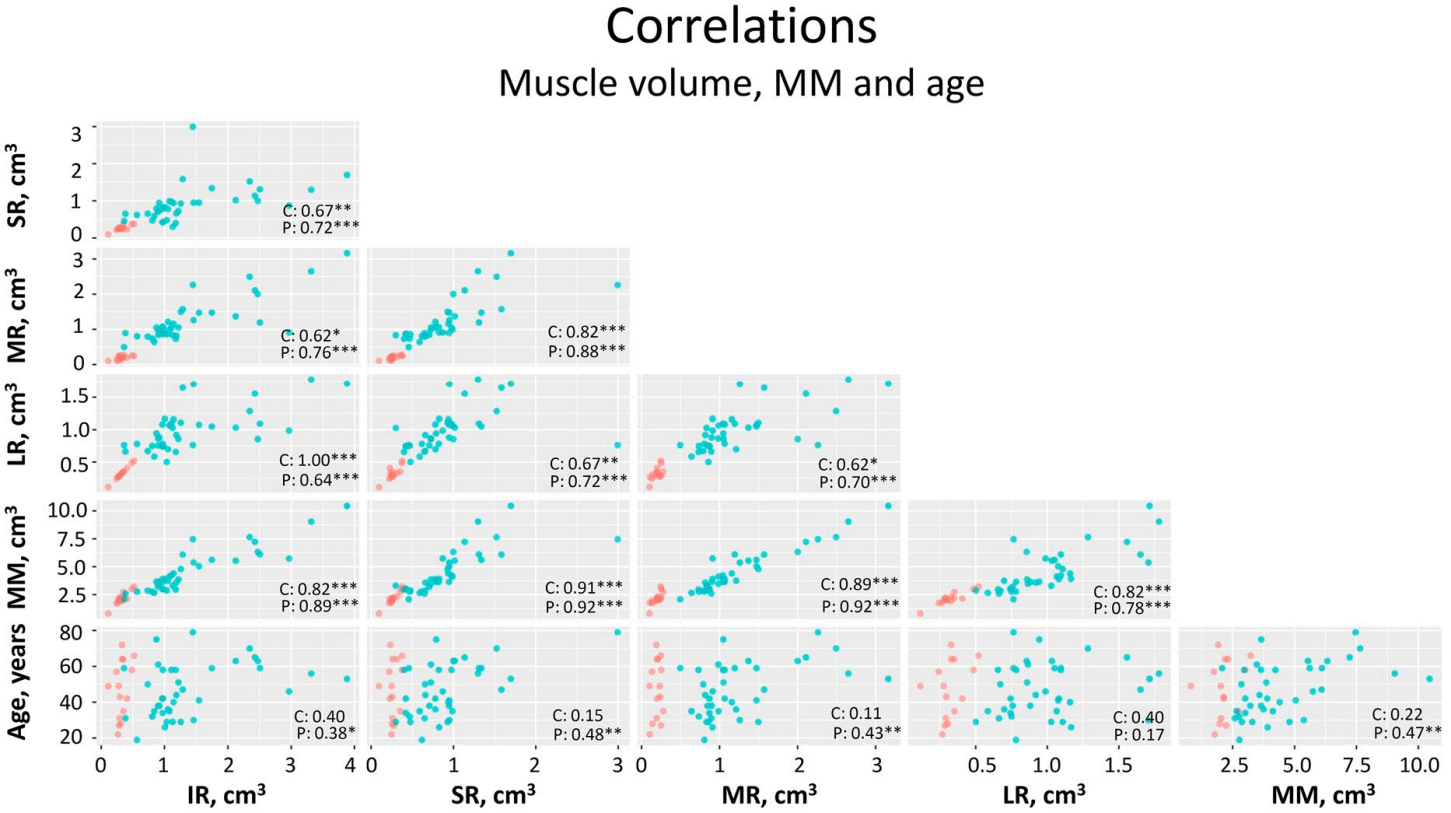
Scatter diagram matrix showing the correlations among the combinations of volume of muscles, MM and age. Control group is shown in red and patient group in green. At the intersection between rows and columns the corresponding scatter plots and R values are shown. Asterisks indicate the statistical significance of p value (no asterisk indicates no significant *p* value; * = 0.05; ** = 0.01; *** = 0.001). Age is expressed in years and muscle volume in cm3. SR: superior rectus; MR: medial rectus; LR: lateral rectus; IR: inferior rectus; MM: total muscle mass; C: control group; P: patients group

The median total muscle mass (MM) was 1.74 cm^3^ greater in patients’ eyes compared to controls (Mann–Whitney test, *p* < 0.001). The thickest muscle in patients with GO was the RI, followed by the RM and RL. No relationship was found between age and muscle thickening in the control group (r = 0.220, *p* = 0.431). However, in patients with GO, a significant positive correlation was observed between MM and age (r = 0.467, *p* < 0.003), being the older the age, the thicker the SR, MR, and IR muscles. The LR is the only muscle for which we have not found this relationship (Fig. 2). As with MM, orbital fat directly correlates with age in patients with GO (r = 0.482; *p* = 0.004) but not in controls (r = 0.422; *p* = 0.117).

When we analyzed differences in optic nerve length between controls and patients with GO, we found that patients had significantly longer optic nerves than controls (Mann–Whitney test; *p* < 0.001). The median optic nerve length in controls was 3.11 cm (range 2.78–4.19), while in the patient group, it was 3.70 cm (range 2.53–4.89).

The CAS score was related to total MM (Kruskal–Wallis test, *p* = 0.013) and orbital fat (Kruskal–Wallis, *p* = 0.048). Likewise, there was a significant relationship between NO-SPECS score and total MM (Kruskal–Wallis test, *p* = 0.029). However, no significant correlation was detected between NO-SPECS score and total orbital fat (Kruskal–Wallis test, *p* = 0.391). High CAS is also related to increased optic nerve length. Thus, for CAS level 3, the median was 3.27 cm (range 2.59–4.64), and for CAS 5, 3.51 cm (range 3.45–3.57).

Lastly, we compared orbital parameters (orbital fat, total MM and volume of each muscle) and serum concentrations of various biomarkers. We observed a positive correlation between serum concentrations of anti-TPO and IR (r = 0.700; *p* = 0.036) thickening. Likewise, a positive correlation was found between ESR values and LR muscle volume (Spearman test 0.473; *p* = 0.013). No significant correlations were found between thyroid hormones (free T4, free T3, TSH) and extraocular muscle volume in patients with GO.

## Discussion

To our knowledge, this is the first study to manually quantify orbital structures in patients with GO and controls using commercial available software and CT or MRI orbital images. In general, our volumetric data were lower than most of the published studies. These differences are probably due to different measurement techniques since most studies used estimated or automated calculations, not real measurements. Despite this, our results agree with previous studies which reported muscle thickening in patients with hyperthyroidism or GO patients (Table 2) [7, 11-16].

**Table 2:** Muscle volume. The mean values in cm^3^ described in the different published series are shown. W: Women; M: Men; GD A: patients with GD with low fat volume/muscle volume ratio; GD B: patients with GD with normal fat volume/muscle volume ratio; R: Right; L: Left

Muscle volume (cm^3^)											
Study		Patients					Controls				
		Muscles					Muscles				
		SR	MR	LR	IR	MM	SR	MR	LR	IR	MM
*Yamamoto *et al						6.57 ± 2.70					3.32 ± 1.31
*Forbes *et al						GD A: 9.09 GD B: 7.1			W: 4.79 ± 0.85 M: 4.52 ± 0.68		
*Feldon *et al		0.59 ± 0.28	0.63 ± 0.28	0.39 ± 0.14	0.59 ± 0.29						
*Forbes *et al						3.80–16.15					W: 3.07–6.80 M: 3.66–6.20
*Troelstra *et al						1.7–12.6					
*Villadolid *et al						3.2 ± 1.2					
*Firbank *et al		0.903 ± 0.35	0.818 ± 0.28	0.689 ± 0.17	0.881 ± 0.41		0.917	0.662	0.692	0.568	
*Nishida *et al		1.22	1.27	0.73	1.37	5.2	0.6	0.49	0.49	0.42	2.43
*Szucs *et al		1.32	0.905	0.775	1.35		0.88	0.79	0.655	0.91	
*Hu *et al		1.7 ± 0.7	1.3 ± 0.6	1.1 ± 0.5	1.3 ± 0.6	5.7 ± 2.4					
*Rodr.-Glez *et al						5.5					4.24
*Potgieser *et al						5.2					
*Lee *et al		R: 0.669			R: 0.919		R: 0.471			R: 0.601	
		L: 0.693			L: 0.979		L: 0.494			L: 0.604	
*Our work*		0.41	0.47	0.47	0.56	3.88	0.23	0.26	0.20	0.31	2.14

Among our patients, the thickest muscle was the IR, followed by the MR and the LR. These results are consistent with those of Nishida et al. [7] and Boncoeur et al. [17]. Villadolid et al. [16] found the IR most frequently affected in patients with GO and GD without apparent ophthalmopathy. Enzmann et al. [12] also observed thickening of IR, MR, SR, and LR (in 77%, 75%, 51% and 50% of cases respectively).

The orbital fat volumes of our patients ranged from 14.2 to 44.19 cm^3^ per orbit. The median total fat observed volume was 44.61 cm^3^ for patients and 38.66 cm^3^ for controls. When separating by orbit, the average fat volumes were 22.3 cm^3^ in patients and 19.33 cm^3^ in controls, similar to prior studies [4, 11]. In our study, both total muscle volume (MM) and total orbital fat volume showed relationship with GO activity. Tachibana et al. [18] observed a positive correlation between muscle volume and disease-related inflammation assessed by CAS. Kvetny et al. [19] observed a significant correlation between extraocular muscle volume and CAS. The large cohort of patients with long-standing inactive GO may explain why other researchers did not observe this relationship. Le Moli et al. [20] also observed a positive correlation between CAS and muscle volume. Byun et al. [21] found that CAS increases with muscle, lacrimal gland, and fat volumes. Comerci et al. [22] also found a significant correlation between orbital fat volume and CAS.

We observed a direct relationship between serum concentrations of anti-TPO antibodies and IR thickening, and also a positive correlation between ESR and LR muscle volume; however, we did not observe an association between muscle volume and TSI antibody levels like that found by other authors [10, 19]. These differences might be attributable to our relatively small study cohort because most patients had less-severe NO-SPECS scale scores.

Our results should be interpreted cautiously since our study was retrospective. Also, given the relatively small size of our cohort, the results we have obtained may not generalize to other populations. In addition, several factors, such as sex and racial characteristics, must be considered when comparing ocular and orbital volumes. The SR alone can be particularly difficult to measure because the resolution of the imaging studies does not allow a reliable isolation from the upper eyelid levator muscle. Also, the volume we measured included vascular and nervous components and this could have caused us to overestimate the volumes of certain structures, mostly orbital fat.

We believe this work represents the first attempt to manually quantify orbital structures in patients with GO using computer aided image analysis and compare them with laboratory test results. Future studies will be required to validate our findings and deepen our understanding of how to best diagnose and treat patients with this challenging disease.

## Supplementary Information

Below is the link to the electronic supplementary material.

Supplementary file1 (DOCX 16 KB) Descriptive summary of ophthalmic examination and serum parameters
