# Supplementary material for: Computer aided volumetric assessment of orbital structures in patients with Graves' orbitopathy: correlation with serum thyroid antiperoxidase antibodies and disease activity
Source: Int Ophthalmol. 2023 Jun 20;43(9):3377–84. doi: 10.1007/s10792-023-02745-8 (PMC10400667; doi:10.1007/s10792-023-02745-8)
Supplement: Supplementary file 1 — Supplementary file1 (DOCX 16 KB) Descriptive summary of ophthalmic examination and serum parameters [file 10792_2023_2745_MOESM1_ESM.docx]

|  | **Group** | |  |  |
| --- | --- | --- | --- | --- |
|  | **Controls** | **Patients** | **p** |  |
| Diplopia (%) | 0% | 25.6% | <0.001 |  |
| Exophthalmia (%) | 0% | 75.6% | <0.001 |  |
| AV (Snellen) | 0.8 (0.6-1.0) | 0.9 (0.4-1.0) | 0.732 |  |
| IOP (mmHg) | 14 (8-22) | 16 (10-23) | 0.696 |  |
| T3 (pg/mL) | 3.01 (1.95-3.59) | 3.22 (2.07-14.61) | 0.156 |  |
| T4 (ng/dL) | 1.14 (0.86-1.87) | 1.23 (0.31-4.45) | 0.248 |  |
| TSH (mIU/L) | 1.95 (0.23-6.72) | 2.12 (0.01-69.30) | 0.897 |  |
| TSI (IU/L) | 0.4 (0.10-0.80) | 4.00 (0.10-40.00) | 0.054 |  |
| AntiTPO (IU/mL) | 33 (28-53) | 221 (22-4521) | 0.057 |  |
| Tg (ng/mL) | 2 (0.5-80) | 1 (0.2-122) | 0.213 |  |
| ESR (mm/h) | 6 (2-19) | 11 (2-34) | 0.139 |  |
